# Statement in Support of Diversity, Equity, and Inclusion for AGU GeoHealth Section

**DOI:** 10.1029/2024GH001297

**Published:** 2024-12-23

**Authors:** K. Ardon‐Dryer, F. Lo, U. Ovienmhada

**Affiliations:** ^1^ Department of Geosciences Texas Tech University Lubbock TX USA; ^2^ Environmental Defense Fund New York NY USA; ^3^ School of Geography, Development, and Environment University of Arizona Tucson AZ USA

**Keywords:** diversity, equity, and inclusion, GeoHealth

## Abstract

This commentary presents the American Geophysical Union's GeoHealth section statement in support of diversity, equity, and inclusion (DEI). The GeoHealth section is an open community that represents diverse backgrounds in the geophysical, biological, and public health sciences that share a passion for research at the nexus of Earth and health sciences. The GeoHealth section will aim to advance our understanding of the interactions between the environment, human health, and well‐being while supporting DEI topics. The GeoHealth Section will ensure a strong and sustained focus on different DEI‐related issues by performing different activities presented in this commentary.

## Key Points

- This is the statement in support of diversity, equity, and inclusion for AGU GeoHealth section
- AGU GeoHealth section aims to advance our understanding of the interactions between the environment, human health, and well‐being

## 1 AGU GeoHealth Section Diversity, Equity, and Inclusion (DEI) Statement

The American Geophysical Union's GeoHealth section aims to advance our understanding of the interactions between the environment, human health, and well‐being. Members of this open community represent diverse backgrounds in the geophysical, biological, and public health sciences and share a passion for research at the nexus of Earth and health sciences.

The GeoHealth section recognizes that climate change and environmental hazards, such as air, water, and soil pollution, and changes in eco‐ and food systems have unequal impacts on the health of people and communities. These unequal impacts are often attributed to legacies of systemic racism, segregation, economic marginalization, colonialism, and other forms of oppression or discrimination. Therefore, the American Geophysical Union's GeoHealth section has established a committee on DEI to demonstrate our value of DEI. In addition to supporting DEI topics of study, we encourage a diverse geoscience workforce that brings a variety of perspectives, backgrounds, experiences, and ideas. This is critical to advancing scientific knowledge, promoting innovation, developing insights, and solving complex problems (Hong & Page, 2004; Medin & Lee, 2012). The inclusion of a diverse knowledge base will improve the ability to communicate information and scientific findings to the public at large. Attention to DEI will also help to promote research on topics of concern to different racial, ethnic, and cultural groups (Huntoon & Lane, 2007).

The GeoHealth DEI committee will prioritize DEI issues in the GeoHealth section activities by facilitating conversations and collaborative projects between disciplines and amplifying a diverse array of geographic, socio‐economic, gendered, ideological, and cultural perspectives from across academia as well as the private and non‐profit sectors. The GeoHealth DEI committee believes this is critical to making meaningful and sustainable changes for marginalized communities that are often disproportionately impacted by global climate change and environmental hazards.

The first task of the GeoHealth DEI committee was to create this DEI statement. The DEI statement received feedback from the large committee members of GeoHealth. It was also open for feedback from members of the GeoHealth community during December 2023. A call for feedback was advertised on the GeoHealth section social media and was sent via email to the GeoHealth members. In January 2024, the DEI committee members incorporated the changes and feedback from GeoHealth at large community members into a finalized set of priorities.

The GeoHealth Section will ensure a strong and sustained focus on different DEI‐related issues by performing the following activities:

- **Amplifying voices of diverse underrepresented scientists**—Our committee aims to increase the voices of diverse underrepresented scientists (based on race, gender, socioeconomic status, disability, country of origin, etc.) who are working in GeoHealth disciplines. To encourage the representation of diverse speakers at the AGU annual conference, our committee will remind session chairs to consider relevant speakers who come from underrepresented backgrounds. Underrepresented scientists and topics related to DEI will also be promoted in the GeoHealth quarterly webinars. The GeoHealth committees will also aim to promote a diversity of individuals and/or groups who work in the GeoHealth field on social media and encourage diverse participation in AGU awards processes.
- **Organizing events at the AGU annual meeting that highlight experts and topics related to DEI, Environmental or Climate Justice, and GeoHealth**. An example of such an event was our AGU23 Town Hall: “The Role of the Geosciences in Environmental Justice: Perspectives from Community Leaders on Purposeful Science.” This event brought together community leaders and researchers to discuss best practices for conducting community engagement projects in the geosciences from project ideation to implementation.
- **Supporting early career scientists with a focus on those who are underrepresented in the GeoHealth community**. The GeoHealth DEI committee, in collaboration with the GeoHealth early career committee, will aim to create a mentoring program pairing early career scientists with senior scientists.
- **Sharing resources and opportunities from organizations related to AGU GeoHealth that promote diversity in the geosciences** including the Earth Science Women's Network (ESWN, 2024), and American Meteorological Society (AMS) Women, a part of AMS Board on Representation, Accessibility, Inclusion, and Diversity (BRAID, 2024).
- **Collaborating with AGU DEI committees and other AGU Sections to integrate efforts and promote diversity across activities**. As part of this effort, our committee will help create and host large DEI sessions at AGU or help promote and understand the state of diversity in the large AGU community (via survey).
- **Collaborating with the AGU GeoHealth Journal to promote special issues on topics related to DEI, Environmental or Climate Justice, and GeoHealth**. The DEI GeoHealth committee will work with the GeoHealth Journal editors to create special issues and promote papers from diverse underrepresented authors.

## References

[gh2592-bib-0001] BRAID . (2024). Board on representation, accessibility, inclusion, and diversity. Retrieved from https://www.ametsoc.org/index.cfm/eec/boards/board‐on‐representation‐accessibility‐inclusion‐and‐diversity‐braid/

[gh2592-bib-0002] ESWN . (2024). Earth Science Women's Network. Retrieved from https://eswnonline.org/

[gh2592-bib-0003] Hong, L. , & Page, S. E. (2004). Groups of diverse problem solvers can outperform groups of high‐ability problem solvers. Proceedings of the National Academy of Sciences of the United States of America, 101(46), 16385–16389. 10.1073/pnas.0403723101 15534225 PMC528939

[gh2592-bib-0004] Huntoon, J. E. , & Lane, M. J. (2007). Diversity in the geosciences and successful strategies for increasing diversity. Journal of Geoscience Education, 55(6), 447–457. 10.5408/1089-9995-55.6.447

[gh2592-bib-0005] Medin, D. L. , & Lee, C. D. (2012). Diversity makes better science. Association for Psychological Science. Retrieved from https://go.nature.com/2EixIJk

